# Supplementary material for: Nano-DMS-MaP allows isoform-specific RNA structure determination
Source: Nat Methods. 2023 Apr 27;20(6):849–59. doi: 10.1038/s41592-023-01862-7 (PMC10250195; doi:10.1038/s41592-023-01862-7)
Supplement: Supplementary file 1 — Supplementary Figs. 1–5. [file 41592_2023_1862_MOESM1_ESM.pdf]

nature methods

Article

https://doi.org/10.1038/s41592-023-01862-7

Nano-DMS-MaP allows isoform-specific RNA structure determination

In the format provided by the authors and unedited

Supplementary Figures, Tables and Legends

Supplementary Figures, Tables and Legends 1

Suppl. Figure 1: Optimization of reverse transcription conditions 2

Suppl. Figure 2: Heat map of 5’UTR DMS reactivities for cell and virion samples..................... 4

Suppl. Figure 3: Specific RT-PCR for the HIV-1 splicing landscape 6

Suppl. Figure 4: Read counts per assignment and isoform 8

Suppl. Figure 5: Mutation distribution in nanopore and Illumina datasets................................ 10

Suppl. Figure 1: Optimization of reverse transcription conditions

(**a**) RT-PCR of a ~500 nt fragment of the HIV-1 unspliced RNA containing the highly structured 5’UTR from HIV-1 expressing cells treated with different concentrations of DMS. (**b**) Optimization of a 5.5 kb RT-PCR from HIV-1 expressing cells by varying DMS concentrations and DMS modification temperature. (**c**) Optimization of a RT-PCR of a complex splicing mixture from HIV-1 expressing cells by varying DMS concentration, Mn^{2+} concentration, and reverse transcription temperature. DNA ladder was 1kb plus (NEB).

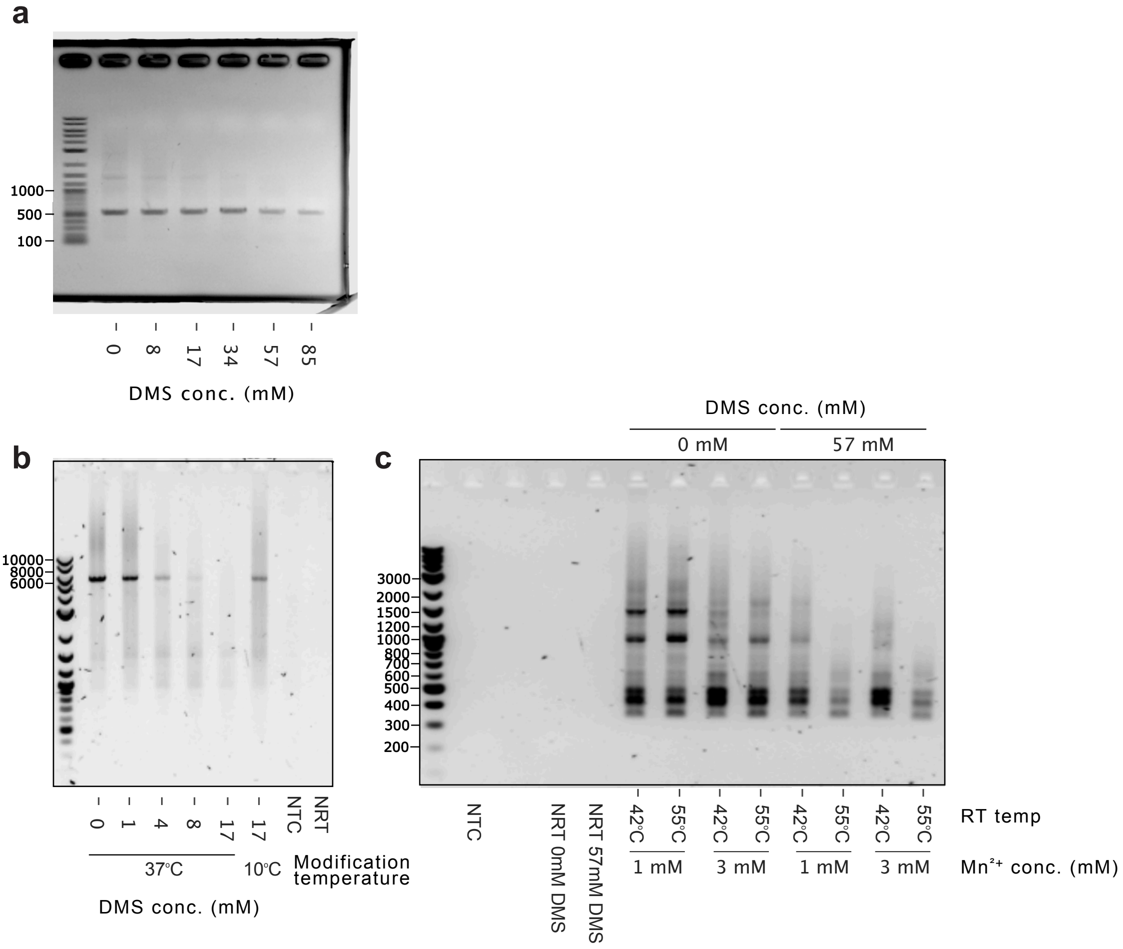
a
1000
500
100
0
8
17
34
57
85
DMS conc. (mM)
b
10000
8000
6000
0
1
4
8
17
17
NTC
NRT
37°C
10°C
Modification temperature
DMS conc. (mM)
c
DMS conc. (mM)
0 mM
57 mM
3000
2000
1500
1200
1000
800
700
600
500
400
300
200
NTC
NRT 0mM DMS
NRT 57mM DMS
42°C
55°C
42°C
55°C
42°C
55°C
42°C
55°C
RT temp
1 mM
3 mM
1 mM
3 mM
Mn²⁺ conc. (mM)

Suppl. Figure 2: Heat map of 5’UTR DMS reactivities for cell and virion samples

Heat map of DMS reactivities for A and C residues for the unspliced HIV-1 RNA found in cells and virions at two DMS concentrations (10 mM and 20 mM). DMS reactivities shown for each DMS concentration is a mean of two independent experiments. The primer binding site (PBS) which binds a cellular tRNA in virions is highlighted. The dimerization initiation site (DIS) which forms an inter-molecular interaction is highlighted.

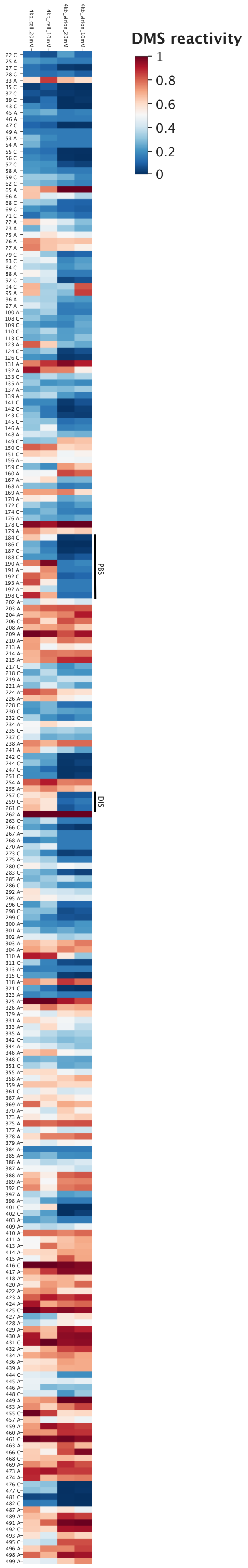
44b_cell_20mM
44b_cell_10mM
44b_virion_20mM
44b_virion_10mM
DMS reactivity
1
0.8
0.6
0.4
0.2
0
PBS
DIS

Suppl. Figure 3: Specific RT-PCR for the HIV-1 splicing landscape

(**a**) RT-PCR-US specifically amplifies unspliced genomic RNA (**b**) RT-PCR-SS specifically amplifies a population of single spliced RNAs. (**c**) RT-PCR-FS specifically amplifies a population of fully spliced RNAs. (**d**) Optimization of the RT-PCR-FS with DNA polymerase Q5 and PrimeSTAR GXL at two different reverse transcription temperatures. DNA ladder for all gels was 1kb plus (NEB). (**e**) Uncropped virtual gel showing diverse species amplified at each DMS concentration for RT-PCR-PS sample.

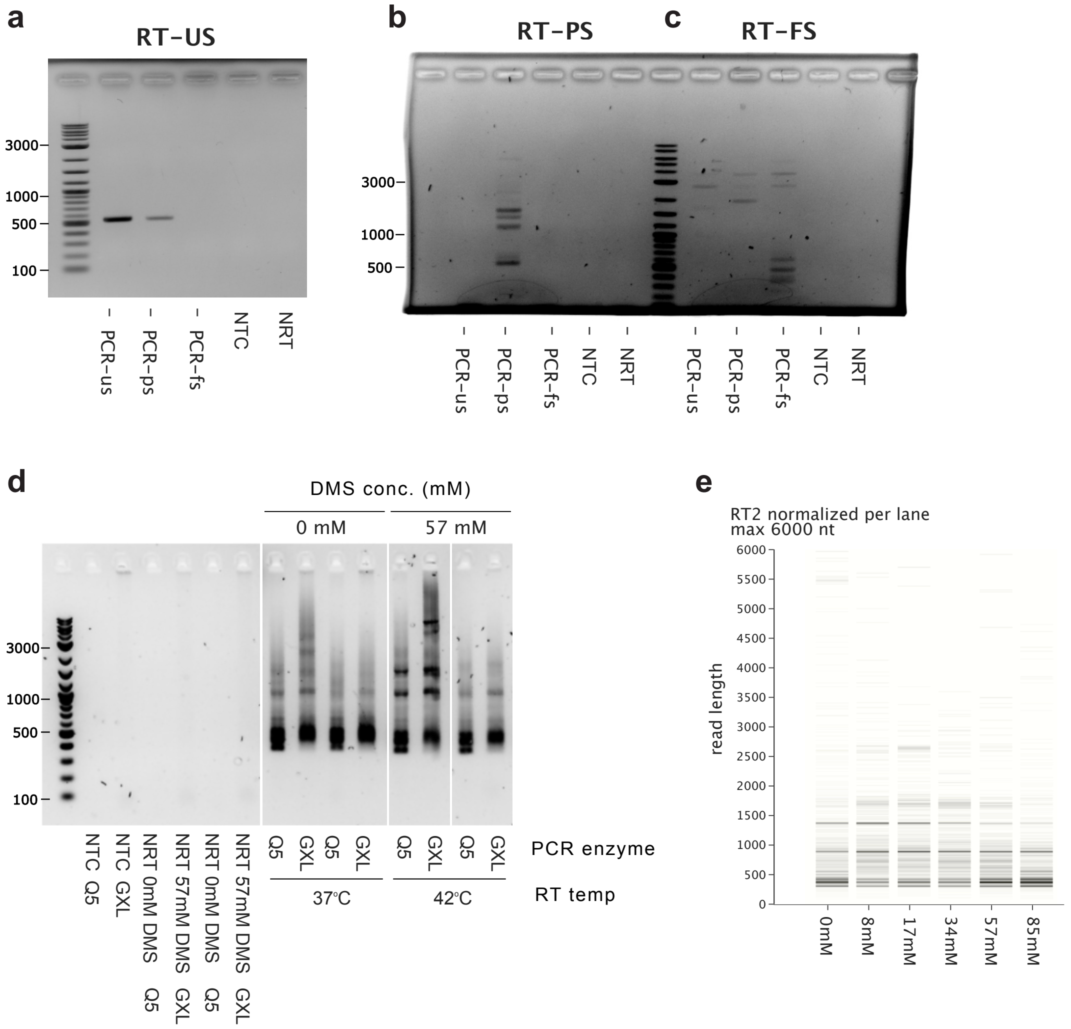
a
RT-US
3000
1000
500
100
PCR-us
PCR-ps
PCR-fs
NTC
NRT
b
RT-PS
c
RT-FS
3000
1000
500
PCR-us
PCR-ps
PCR-fs
NTC
NRT
PCR-us
PCR-ps
PCR-fs
NTC
NRT
d
DMS conc. (mM)
0 mM
57 mM
3000
1000
500
100
NTC Q5
NTC GXL
NRT 0mM DMS Q5
NRT 57mM DMS GXL
NRT 0mM DMS Q5
NRT 57mM DMS GXL
Q5
GXL
Q5
GXL
Q5
GXL
Q5
GXL
PCR enzyme
37°C
42°C
RT temp
e
RT2 normalized per lane
max 6000 nt
read length
6000
5500
5000
4500
4000
3500
3000
2500
2000
1500
1000
500
0
0mM
8mM
17mM
34mM
57mM
85mM

Suppl. Figure 4: Read counts per assignment and isoform

(**a**) Representative data from a single nanopore sequencing run showing read count per assignment IsoQuant type for RT-PCR-US, RT-PCR-PS and RT-PCR-FS. “Unique” are sequencing reads mapping uniquely to a transcript isoform. “Unique minor differences” reads map to a single isoforms, but with alignment artifacts. Ambiguous reads are compatible with matching to more than one transcript. Inconsistent reads map with non-intronic inconsistencies (e.g., alternative transcription or polyadenylation sites). Non-informative reads are unmapped reads. (**b**) Representative data from a single nanopore sequencing run showing read counts per isoform assignment for RT-PCR-US, RT-PCR-PS and RT-PCR-FS as determined by IsoQuant. (**c**) Sunburst plots showing relative expression of each transcript grouped by isoform (left) or first acceptor site (right).

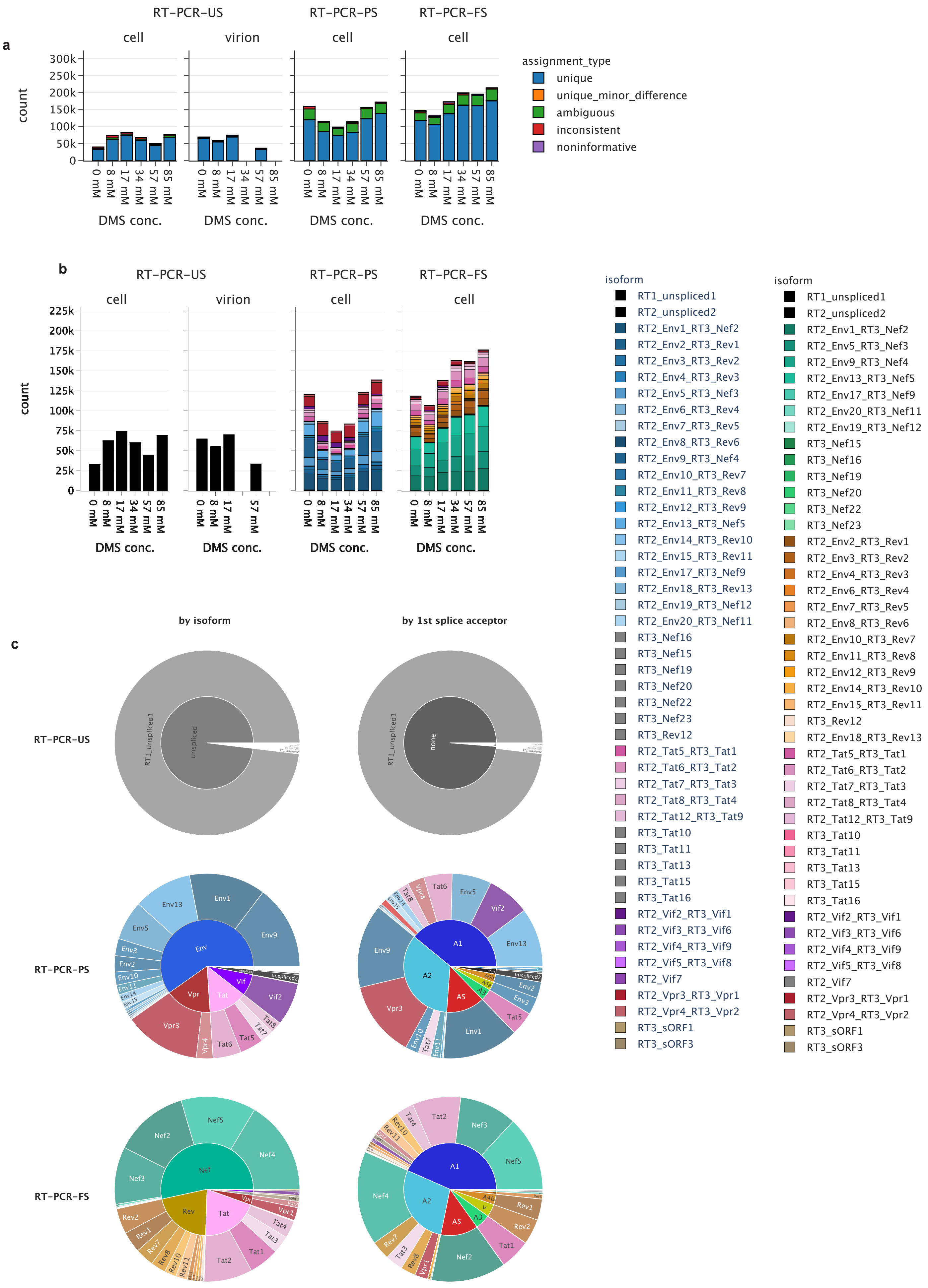
a
RT−PCR−US
RT−PCR−PS
RT−PCR−FS
cell
virion
count
300k
250k
200k
150k
100k
50k
0
0 mM
8 mM
17 mM
34 mM
57 mM
85 mM
DMS conc.
assignment_type
unique
unique_minor_difference
ambiguous
inconsistent
noninformative
b
225k
200k
175k
150k
125k
100k
75k
50k
25k
isoform
RT1_unspliced1
RT2_unspliced2
RT2_Env1_RT3_Nef2
RT2_Env2_RT3_Rev1
RT2_Env3_RT3_Rev2
RT2_Env4_RT3_Rev3
RT2_Env5_RT3_Nef3
RT2_Env6_RT3_Rev4
RT2_Env7_RT3_Rev5
RT2_Env8_RT3_Rev6
RT2_Env9_RT3_Nef4
RT2_Env10_RT3_Rev7
RT2_Env11_RT3_Rev8
RT2_Env12_RT3_Rev9
RT2_Env13_RT3_Nef5
RT2_Env14_RT3_Rev10
RT2_Env15_RT3_Rev11
RT2_Env17_RT3_Nef9
RT2_Env18_RT3_Rev13
RT2_Env19_RT3_Nef12
RT2_Env20_RT3_Nef11
RT3_Nef16
RT3_Nef15
RT3_Nef19
RT3_Nef20
RT3_Nef22
RT3_Nef23
RT3_Rev12
RT2_Tat5_RT3_Tat1
RT2_Tat6_RT3_Tat2
RT2_Tat7_RT3_Tat3
RT2_Tat8_RT3_Tat4
RT2_Tat12_RT3_Tat9
RT3_Tat10
RT3_Tat11
RT3_Tat13
RT3_Tat15
RT3_Tat16
RT2_Vif2_RT3_Vif1
RT2_Vif3_RT3_Vif6
RT2_Vif4_RT3_Vif9
RT2_Vif5_RT3_Vif8
RT2_Vif7
RT2_Vpr3_RT3_Vpr1
RT2_Vpr4_RT3_Vpr2
RT3_sORF1
RT3_sORF3
isoform
RT1_unspliced1
RT2_unspliced2
RT2_Env1_RT3_Nef2
RT2_Env5_RT3_Nef3
RT2_Env9_RT3_Nef4
RT2_Env13_RT3_Nef5
RT2_Env17_RT3_Nef9
RT2_Env20_RT3_Nef11
RT2_Env19_RT3_Nef12
RT3_Nef15
RT3_Nef16
RT3_Nef19
RT3_Nef20
RT3_Nef22
RT3_Nef23
RT2_Env2_RT3_Rev1
RT2_Env3_RT3_Rev2
RT2_Env4_RT3_Rev3
RT2_Env6_RT3_Rev4
RT2_Env7_RT3_Rev5
RT2_Env8_RT3_Rev6
RT2_Env10_RT3_Rev7
RT2_Env11_RT3_Rev8
RT2_Env12_RT3_Rev9
RT2_Env14_RT3_Rev10
RT2_Env15_RT3_Rev11
RT3_Rev12
RT2_Env18_RT3_Rev13
RT2_Tat5_RT3_Tat1
RT2_Tat6_RT3_Tat2
RT2_Tat7_RT3_Tat3
RT2_Tat8_RT3_Tat4
RT2_Tat12_RT3_Tat9
RT3_Tat10
RT3_Tat11
RT3_Tat13
RT3_Tat15
RT3_Tat16
RT2_Vif2_RT3_Vif1
RT2_Vif3_RT3_Vif6
RT2_Vif4_RT3_Vif9
RT2_Vif5_RT3_Vif8
RT2_Vif7
RT2_Vpr3_RT3_Vpr1
RT2_Vpr4_RT3_Vpr2
RT3_sORF1
RT3_sORF3
c
by isoform
by 1st splice acceptor
RT−PCR−US
RT−PCR−PS
RT−PCR−FS
RT1_unspliced1
unspliced
none
Env
Env1
Env13
Env9
Env5
Env3
Env2
Env10
Env11
Env14
Env15
Vpr
Vpr3
Vpr4
Tat
Tat5
Tat6
Tat7
Tat8
Vif
Vif2
unspliced2
A1
A2
A5
A3
Nef
Nef2
Nef3
Nef4
Nef5
Rev
Rev1
Rev2
Rev7
Rev8
Rev10
Rev11
Tat1
Tat2
Tat3
Tat4
Vpr1

Suppl. Figure 5: Mutation distribution in nanopore and Illumina datasets

Histograms of per read mutation count distributions (left), per read mutation percentages (middle) and per read aligned read length (right) as saved in RNA-Framework's mutation map (mm) files shown for (**a**) nanopore and (**b**) Illumina datasets obtained from matched samples of the unspliced (US) HIV-1 RNA probed at different DMS concentrations (rows). Data for independent experimental replicates are shown separately (blue and grey bars). Reads with 0 mutations are excluded from this analysis.

a

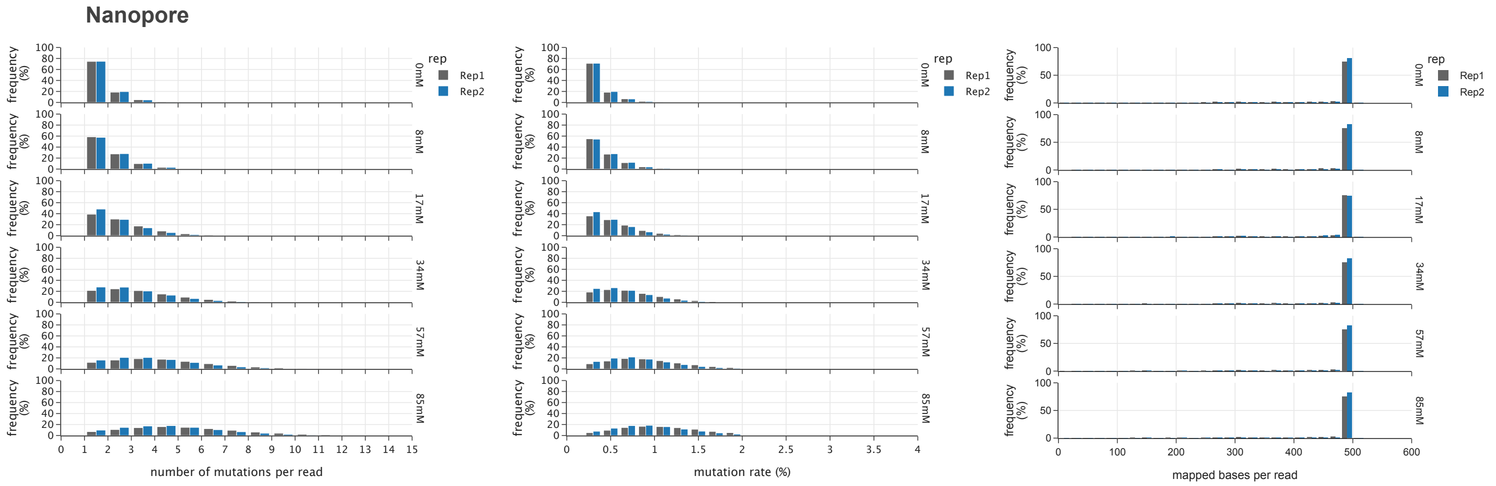
Nanopore
frequency (%)
rep
Rep1
Rep2
0mM
8mM
17mM
34mM
57mM
85mM
number of mutations per read
mutation rate (%)
mapped bases per read

b

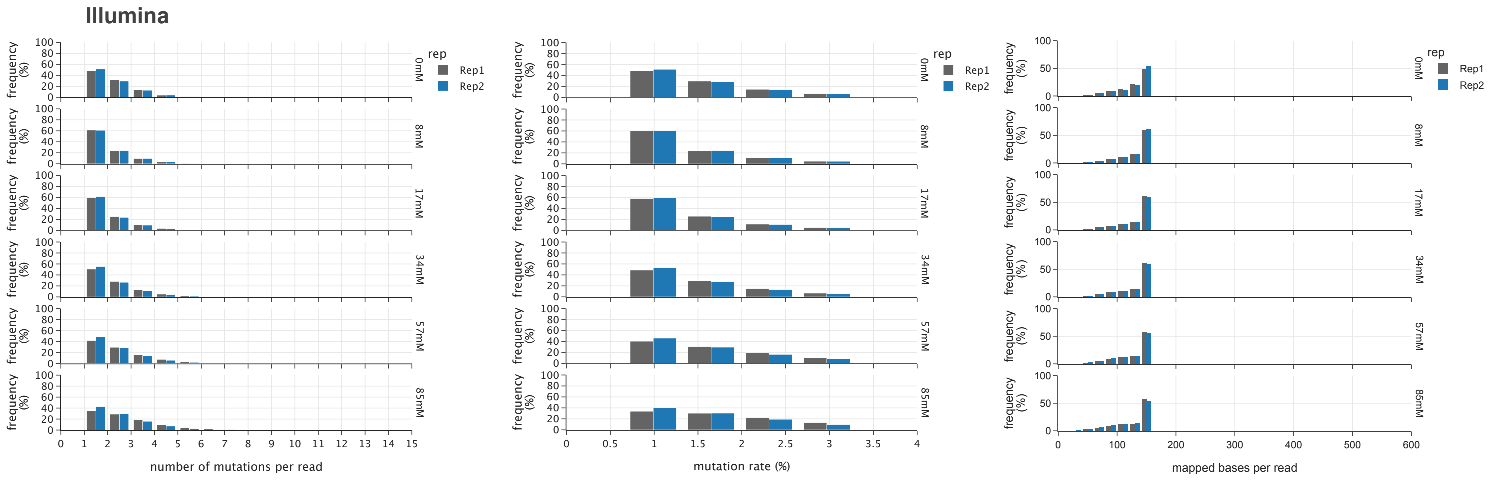
Illumina
frequency (%)
rep
Rep1
Rep2
0mM
8mM
17mM
34mM
57mM
85mM
number of mutations per read
mutation rate (%)
mapped bases per read
