# Supplementary material for: Nano-DMS-MaP allows isoform-specific RNA structure determination
Source: Nat Methods. 2023 Apr 27;20(6):849–59. doi: 10.1038/s41592-023-01862-7 (PMC10250195; doi:10.1038/s41592-023-01862-7)
Supplement: Supplementary file 2 — Reporting Summary [file 41592_2023_1862_MOESM2_ESM.pdf]

nature research

Corresponding author(s): Smyth RP

Last updated by author(s): Feb 27, 2023

Reporting Summary

Nature Research wishes to improve the reproducibility of the work that we publish. This form provides structure for consistency and transparency in reporting. For further information on Nature Research policies, see our Editorial Policies and the Editorial Policy Checklist.

Statistics

For all statistical analyses, confirm that the following items are present in the figure legend, table legend, main text, or Methods section.

n/a	Confirmed	
☐	☒	The exact sample size (*n*) for each experimental group/condition, given as a discrete number and unit of measurement
☐	☒	A statement on whether measurements were taken from distinct samples or whether the same sample was measured repeatedly
☒	☐	The statistical test(s) used AND whether they are one- or two-sided<br>*Only common tests should be described solely by name; describe more complex techniques in the Methods section.*
☒	☐	A description of all covariates tested
☒	☐	A description of any assumptions or corrections, such as tests of normality and adjustment for multiple comparisons
☐	☒	A full description of the statistical parameters including central tendency (e.g. means) or other basic estimates (e.g. regression coefficient) AND variation (e.g. standard deviation) or associated estimates of uncertainty (e.g. confidence intervals)
☒	☐	For null hypothesis testing, the test statistic (e.g. *F*, *t*, *r*) with confidence intervals, effect sizes, degrees of freedom and *P* value noted<br>*Give P values as exact values whenever suitable.*
☒	☐	For Bayesian analysis, information on the choice of priors and Markov chain Monte Carlo settings
☒	☐	For hierarchical and complex designs, identification of the appropriate level for tests and full reporting of outcomes
☐	☒	Estimates of effect sizes (e.g. Cohen's *d*, Pearson's *r*), indicating how they were calculated

Our web collection on statistics for biologists contains articles on many of the points above.

Software and code

Policy information about availability of computer code

Data collection	Data was generated on Oxford Nanopore Technologies Minion Mk1B using Minknow software version (21.11.8) and a R10.4 MinIon flow cell (FLO-MIN112).
Data analysis	Data was basecalled with guppy 6.1.3. Virtual gels were generated with a custom python v3.8.5 script using the numpy library version 1.19.2. Read to isoform mapping was performed using IsoQuant version 2.0. Alignments were performed on specific reference sequences using LAST version 1419. sam files were then processed using samtools version 1.12. Mismatch patterns were analyzed from BAM alignment files with perbase version 0.8.3 and custom python scripts. Mutational Profiling analysis was performed for each isoform separately using RNAFramework version 2.7.2 or custom python scripts. Correlation scores of reactivity profiles was calculated using the python package scikit-learn version 0.21.3. Sub-figures were generated using the python library plotly version 4.14.3. All custom scripts will be supplied before publication on GitHub (https://github.com/smyth-lab/Nano-DMS-MaP). De novo RNA structure prediction was performed with Eternafold version 1.3.1. RNA structures were visualised using VARNA version 3.93.

For manuscripts utilizing custom algorithms or software that are central to the research but not yet described in published literature, software must be made available to editors and reviewers. We strongly encourage code deposition in a community repository (e.g. GitHub). See the Nature Research guidelines for submitting code & software for further information.

Data

Policy information about availability of data

All manuscripts must include a data availability statement. This statement should provide the following information, where applicable:

- Accession codes, unique identifiers, or web links for publicly available datasets
- A list of figures that have associated raw data
- A description of any restrictions on data availability

All basecalled data are available on the sequence read archive (SRA) at Bioproject accession number PRJNA938445 and Sequencing Project Number SRP424422

Field-specific reporting

Please select the one below that is the best fit for your research. If you are not sure, read the appropriate sections before making your selection.

☒ Life sciences ☐ Behavioural & social sciences ☐ Ecological, evolutionary & environmental sciences

For a reference copy of the document with all sections, see nature.com/documents/nr-reporting-summary-flat.pdf

Life sciences study design

All studies must disclose on these points even when the disclosure is negative.

Sample size	We performed two biologically independent experimental replicates. This sample size was chosen because of previously known high correlation between biological replicates.
Data exclusions	All reads with a mean read quality score below 10 and those not demultiplexed by the basecaller were removed, as described in the manuscript.
Replication	We assessed reproducibility by calculating Pearson's correlation coefficient between two experimental replicates for relative abundance of transcript isoforms and for DMS reactivities. All replications were successful, and indicated in the manuscript.
Randomization	Randomisation was not performed as it was not relevant to our study.
Blinding	Blinding was not performed as it was not relevant to our study.

Reporting for specific materials, systems and methods

We require information from authors about some types of materials, experimental systems and methods used in many studies. Here, indicate whether each material, system or method listed is relevant to your study. If you are not sure if a list item applies to your research, read the appropriate section before selecting a response.

Materials & experimental systems

n/a	Involved in the study	
☒	☐	Antibodies
☐	☒	Eukaryotic cell lines
☒	☐	Palaeontology and archaeology
☒	☐	Animals and other organisms
☒	☐	Human research participants
☒	☐	Clinical data
☒	☐	Dual use research of concern

Methods

n/a	Involved in the study	
☒	☐	ChIP-seq
☒	☐	Flow cytometry
☒	☐	MRI-based neuroimaging

Eukaryotic cell lines

Policy information about cell lines

Cell line source(s)	HEK 293T cells were obtained from the Caliskan laboratory.
Authentication	HEK 293T cells were not independently authenticated.
Mycoplasma contamination	HEK 293T cells are tested for mycoplasma infection monthly, and were consistently negative.
Commonly misidentified lines (See ICLAC register)	N/A
