# Supplementary figures and images for: Nano-DMS-MaP allows isoform-specific RNA structure determination
Source: Nat Methods. 2023 Apr 27;20(6):849–59. doi: 10.1038/s41592-023-01862-7 (PMC10250195; doi:10.1038/s41592-023-01862-7)

Uncropped gels. Fig 4c

Fig. 4c

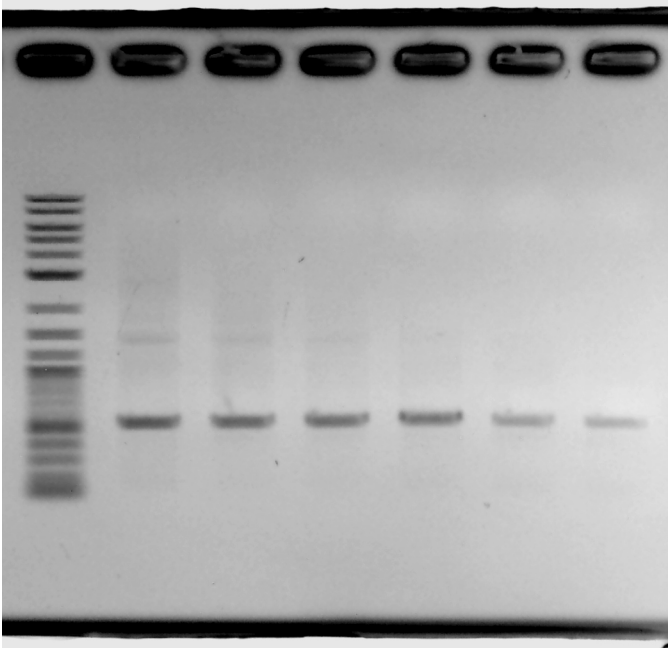

Supplement: Supplementary file 7 — Uncropped gels for main Fig. 4c. [file 41592_2023_1862_MOESM7_ESM.pdf]

Uncropped gels. Fig 4d

Fig. 4d

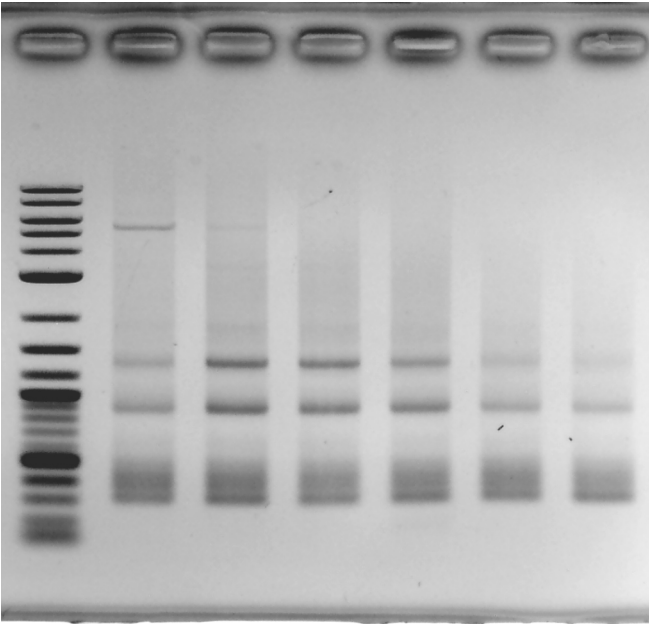

Supplement: Supplementary file 8 — Uncropped gels for main Fig. 4d. [file 41592_2023_1862_MOESM8_ESM.pdf]

Uncropped gels. Fig 4e

Fig. 4e

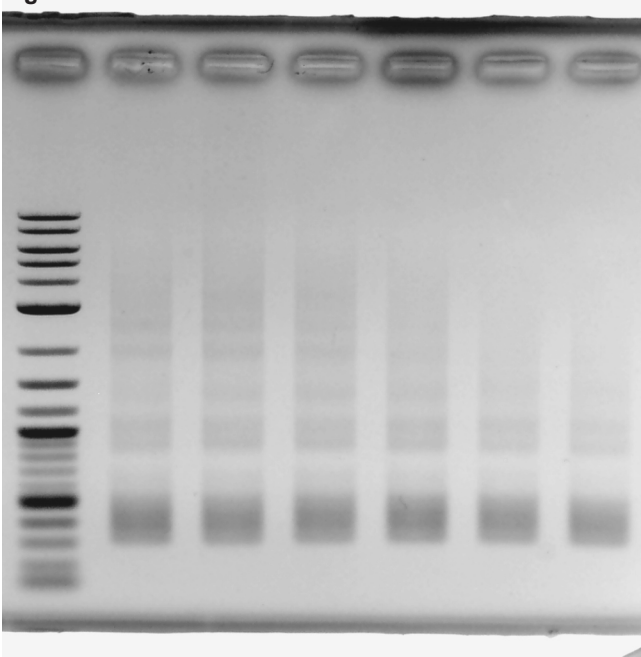

Supplement: Supplementary file 9 — Uncropped gels for main Fig. 4e. [file 41592_2023_1862_MOESM9_ESM.pdf]
